# Assessment of Psychometric Properties of the Malay Version of the Brief Resilience Scale (BRS-M) among Non-Academic Staff Working from Home during COVID-19 in Malaysia

**DOI:** 10.3390/healthcare11081146

**Published:** 2023-04-17

**Authors:** Zuraida Ahmad Sabki, Lee Hui Kim, Mahmoud Danaee, Ahmad Hatim Sulaiman, Khairul Arif Razali, Ong Hui Koh, Sharmilla Kanagasundram, Manveen Kaur, Fatin Liyana Azhar, Benedict Francis

**Affiliations:** 1 Department of Psychological Medicine, Faculty of Medicine, University of Malaya, Kuala Lumpur 50603, Malaysia; 2 Department of Social and Preventive Medicine, Faculty of Medicine, University of Malaya, Kuala Lumpur 50603, Malaysia; 3 Department of Psychological Medicine, University Malaya Medical Centre, Lembah Pantai, Kuala Lumpur 59100, Malaysia

**Keywords:** psychometric properties, Malay BRS, resilience, burnout, working from home, non-academic staff

## Abstract

This study aims to validate the Malay version of the Brief Resilience Scale (BRS-M) in order for the scale to be available among the Malay-speaking population. Two hundred and ninety-eight non-academic staff completed the Malay version of the Brief Resilience Scale (BRS-M), Malay Copenhagen Burnout Inventory (CBI-M), and Malay Depression, Anxiety, and Stress Scale (M-DASS-21). To explore the factor structure of BRS-M, exploratory factor analysis (EFA) with the first group of 149 participants was conducted using FACTOR (v.11) software. Confirmatory factor analysis (CFA) was conducted from the data of the second group of 149 participants using SEM_PLS software. The EFA revealed a two-factor model; Factor 1 =”Resilience” and Factor 2 = ”Succumbing”. The CFA indicated a sufficient internal consistency reliability (Cronbach’s α = 0.806 and McDonald’s omega, ω = 0.812) and a good fit with SRMR = 0.031. BRS-M, CBI-M, and M-DASS-21 displayed a satisfactory concurrent validity result. Household income and marital status had significant association with resilience level, with low household income (B40 group) being a predictor of lower resilience. The BRS-M demonstrated favourable psychometric properties in terms of reliability and validity to assess the level of resilience among non-academic staff in Malaysia.

## 1. Introduction

Studies have reported that following COVID-19 pandemic, there was a drastic increase in prevalence of anxiety, depression, and sleep deprivation in the general community, especially those with pre-existing mental disorders [1,2]. According to a survey of 1210 respondents from 194 cities in China, 16.5% reported experiencing symptoms of depression, 28.8% reported anxiety symptoms, and stress was reported in 8.1%, with most rated as moderate to severe symptoms [3]. Overall, 75.2% were worried about infecting their loved ones with COVID-19 [3]. The Malaysian government had to impose a movement control order (MCO) or “partial lockdown” in different phases throughout the pandemic between 2020–2022, forcing the shutting down of many factories, and administrative work to be conducted remotely from home [4]. Globally, there was an exponential increase in the number of workers having to adapt to working from home (WFH), and this trend has persisted post pandemic [5].

Higher education institutions have gone through a significant transformation following the COVID-19 pandemic and are seen as a catalyst for the academician and administrator to shift the traditional approach to teaching and learning for the students toward virtual platforms [6]. Simultaneously, administrative personnel or non-academic staff were notified of WFH by the universities and expected to maintain the same work ethics, productivity, and efficiency. A study that was conducted locally among higher education institutions employees found that a conducive workplace environment at home determined motivation and hence productivity when working from home during the MCO [7]. WFH reduced traveling expenditure and traffic jams, saving costs on food and providing better personal and family life fulfilment [8].

Undoubtedly, WFH affects employees’ work engagement both positively and negatively. Many have encountered challenges in learning to use the latest applications such as “Zoom” and “Google Meet”. It was challenging to rapidly resolve issues through a virtual conversation online, leading to “zoom-fatigue”. Furthermore, the sudden need to perform several duties at the same time while working from home resulted in mental and emotional stress. Many people consequently began to experience job burnout and other detrimental effects [9]. Social isolation, shown by a lack of face-to-face interaction between co-workers, thus disrupts communication, and these disruptions in turn may affect employees’ work styles and team dynamics, leading to negative psychological effects, even though this style of work is essential for halting the COVID-19 spread [10]. Online virtual communication has also been demonstrated to heighten anxiety and depressive symptoms, as employees had to compromise family time for work. Work–family conflict was more likely in WFH employees who had dual responsibilities for caring for their family tasks [11]. As opposed to regular office jobs, employees who WFH were found to spend more time on their tasks without realising it, leading to longer working hours [12]. As a result, they spent less time with their families and had more work–family conflicts.

Various studies have identified factors that function as a buffer against adversities, and these factors are described by the term resilience [13,14,15]. Resilience needs to be viewed as a dynamic process involving positive coping and adaptation [16]. This adaptation can either result in a return to the prior equilibrium state or in a change to another state known as transformation, which enhances resilience further, leading to growth and sustainability [17]. Resilient employers were found to be able to maintain or return to their optimum level of functioning following adverse events [15]. This factor is pertinent in the higher education workforce, where local and global rankings matter the most. It is sufficient to note that non-academic staff provide essential administrative and technical support that the universities need to conduct and sustain academic (e.g., research and teaching activities) and non-academic activities.

Resilience has received increasing interest in respect to its effect on general wellbeing and quality of life [18], leading many organizations to include policies and practices that create resilience among their workers. Resilience-focused intervention to enhance wellness in the workplace requires a standardized evaluation tool to ensure reliable and valid data quality. Resilience has a complex construct that carries different operational definitions such as climate or ecological [19], neurolinguistic [20], spiritual [15], and personal [21] resilience. There has been no instrument that has been considered as a gold standard in the measurement of level of resilience. However, the Brief Resilience Scale was found to be among the few scales that assessed the ability to recover from a stressful event, termed as “the ability to bounce back”, as the outcome measure [13]. BRS, which is a self-administered questionnaire, has been used in both non-clinical and clinical populations and translated into Korean [22], Chinese [23], Spanish [24], Turkish [25], and other languages. Although BRS has been assessed among university students in Malaysia, it has not been translated and validated in the Malay language [26].

The principal aim of this study is to assess the psychometric properties of the Malay version of BRS (BRS-M) and to determine the factor structure. The original BRS demonstrated a single-factor structure based on exploratory factor analysis (EFA) without studying the dimensionality using confirmatory factor analysis (CFA) [27]. For this study, the CFA will test the hypothesized factor structure extracted from the EFA at a different time and with a different group of non-academic staff during the study period. This study includes an assessment of the instrument’s concurrent validity based on previous study that demonstrated a negative correlation between resilience and burnout [28]. Hence, screening any employee with low resilience may reveal important potential mental health issues that require further intervention by the organization. This is pertinent since mental health issues are still highly stigmatised, and BRS can be utilized as a preliminary screening tool before proper assessment using other well-established psychological screening tools.

The second aim is to assess the association between the sociodemographic factors and working environment included in this study, such as working from home, marital status, household income, and gender, with level of resilience using BRS-M. Studies reported a significant association between psychological distress and level of resilience [29,30]. We hypothesized that one’s level of resilience could be affected by age, education background, marital status, and household income.

## 2. Materials and Methods

### 2.1. Study Design

Phase 1: Forward-Backward Translation of BRS.

The BRS was translated from English into the Malay language by a psychiatrist and a linguistic expert. Once the discrepancies were resolved, it was translated back to English from Malay by a counsellor and a licensed translator. Both the translated and original English versions were reviewed by the expert panel committee. The forward-backward translation approach was in accordance with the translation guideline [31]. The questionnaire was pilot-tested among 15 administrative staff before data collection, and all 15 participants agreed that each item was relevant, unambiguous, easy to answer, non-judgmental, and did not cause any distress. A senior clinical psychologist reviewed the finalized Malay version of BRS (BRS-M) for its experiential, idiomatic, semantic, and conceptual equivalence.

Phase 2: Participants, settings, and duration of study.

The study took place between April 2021 and March 2022. The study protocol was approved by the University Malaya Research Ethics Committee (UMREC) (UM. TNC2/UMREC_1308). The approval for translation of the original BRS to the Malay language was received from the original author of Brief Resilience Scale, Bruce Smith [32]. The data obtained from this study were strictly confidential, participation was voluntary, and no remuneration was provided.

The inclusion criteria were the following:

1. 18 years old and above;
2. Consented to participate in the study;
3. Non-academic staff (defined as non-teaching or administrative staff appointed by the university);
4. Able to understand Malay language, as all the questionnaires were in Malay.

The sample size required was based on a 10:1 subject-to-item ratio, and therefore, a minimum of 60 respondents were required [33].

Phase 2a: Group 1 for Exploratory Factor Analysis (EFA) study.

During the period of MCO, most of the non-academic staff were instructed to work from home, while a few remained for on-site work depending on the critical needs of the service. A total of six weekly webinar series were organized by the Human Resource department, with CME points given to encourage participation. We decided to recruit the first group from the first three webinar series using a purposive sampling method. A total of 149 non-academic staff agreed to participate in the study and fill out questionnaires via Google forms. The nature and aims of the study were explained.

Phase 2b: Group 2 for Confirmatory Factor Analysis (CFA) study.

A total of 149 non-academic staff who attended the last three webinar series and had not participated in the first group were recruited and similarly answered the questionnaires via Google forms.

### 2.2. Instruments

#### 2.2.1. Sociodemographic Questionnaire

The sociodemographic profile, including age, ethnic, gender, marital status, education level, and household income, were included in the questionnaire. The sub-categories of household income were top 20% (T20), middle 40% (M40), and bottom 40% (B40). For this study, the household income was categorized as above MYR 9619 (top 20%), MYR 4360–MYR 9619 (middle 40%), and less than MYR 4360 (below 40%) [34]. Relevant questions pertaining to current work arrangement were included, and those who answered “Yes” to be currently working from home were required to assess their level of stress as with options of “No stress”, “Some degree of stress”, or “Significantly stressful”.

#### 2.2.2. Brief Resilience Scale (BRS) and the Malay Version of the Brief Resilience Scale (BRS-M)

BRS was designed as a six-item, self-administered instrument. It has three items that are positively and three items that are negatively framed to reduce response bias [32]. This instrument evaluates a person’s resilience level, defined as “the ability to bounce back following a stressful event”. The scoring is based on a 5-point Likert scale, from 1 (“strongly disagreeing”) to 5 (“strongly agreeing”). Positively worded items are items 1, 3, and 5, and the negatively phrased items are items 2, 4, and 6, and they are scored by reversing their coding, and the mean of the six items is calculated for a final score, which can be in the range of 1 to 5, with a higher score reflecting more resilience. Smith et al. suggested that resilience scores less than 3.00 be construed as low and scores greater than 4.30 as higher resilience. [35]. The Cronbach’s alpha is 0.91 and EFA revealed a one-factor structure, with loadings ranging from 0.68 to 0.91 [32]. Items included in the BRS-M questionnaire were taken from the BRS for validation studies.

#### 2.2.3. Malay Version of Copenhagen Burnout Inventory (CBI-M)

The scale [36] was translated to Malay, and it measures burnout based on three areas, namely work-related burnout (seven items), personal burnout (six items), and client-related burnout (six items) [37]. All items are rated on a 5-point scale (0 = always/to a very high degree, 4 = never/almost never/to a very low degree) [36]. The scale labels are re-coded reversely to 0 (never/almost never and to a very low degree) and 100 (always and to a very high degree) [36]. Therefore, the higher scores indicate increased burnout. The internal consistency was good, with Cronbach’s alpha values of 0.87.

#### 2.2.4. Malay Version of Depression, Anxiety, Stress Scale-21 (M-DASS-21)

The scale has seven items in each scale [38] and was translated to Malay (M-DASS-21) using a 4-point severity/frequency scale to assess how much the respondent experienced the three domains of depression, anxiety, and stress in the previous week [39]. Each domain’s overall scores represent the severity of the respective domains. The Cronbach’s alpha values for the M-DASS-21 were 0.84, 0.74, and 0.79, respectively, for depression, anxiety, and stress [39].

### 2.3. Statistical Analyses

The descriptive statistics were computed using Statistical Package for the Social Sciences version 26.0 (SPSS, Chicago, IL, USA). The normality of data was assessed using the skewness and kurtosis methods [40].

Data from group 1 (n = 149) were analysed for exploratory factor analysis (EFA). A viable factor structure was obtained from the Bartlett’s test of sphericity and the Kaiser–Mayer–Olkin measure of sampling adequacy. The internal consistency of BRS-M and subscales of BRS-M is based on the Cronbach’s alpha. The data in this study were categorical variables, and therefore, the EFA was employed using a polychoric correlation matrix. When both observed variables were dichotomous, the tetrachoric correlation was applied as a special case of the polychoric correlation. When the ordinal item univariate distributions were asymmetric or had an excess of kurtosis, polychoric correlation was advised. It was suggested to use Pearson correlation if the absolute values of both indices were less than one. The factor analysis model for binary variables was applied [41]. Principal components analysis (PCA) and promin rotation were employed, which enabled analyses using a polychoric correlation matrix. Parallel analysis was performed to decide how many factors to retain in the scale [42]. FACTOR software Ver 11.05.01 (2021, Rovira i Virgili University, Tarragona, Spain) for Pratt’s importance measure matrix was used for deciphering multidimensional oblique factor models in the exploratory stage. The data were also used to study the concurrent (criterion) validity between BRS-M and CBI-M and M-DASS-21.

The dimensionality of the factors was obtained from the data in group 2 (n = 149) using confirmatory factor analysis (CFA) and were constructed on partial least square and PLS-SEM method using Smart_PLS Ver 3.3.5 [43]. The PLS-SEM method assessed the reflective measurement models, and the relevant indicators were average variance extracted (AVE) to the assess convergent validity (>0.5) and outer loading (>0.5) output. The reliability of instrument was evaluated based on composite reliability (>0.7), Cronbach’s α (>0.7) [44], and McDonald’s omega (ω) coefficients [45]. Discriminant validity also was tested using the HTMT method (Hetrotrait-Monotrait ratio of criteria; <0.85), followed by checking the cross-loading values [46]. The fit index was ascertained based on the standardized root mean square residual (SRMR) with the threshold for acceptable model fit of SRMR≤0.08 [47].

The associations between sociodemographic data and the factor scores were established through the analysis of variance and variables, with significant relationships included in the multivariable analysis.

## 3. Results

A total of 298 non-academic staff were recruited from the two groups, and 98% were working from home during the study period. Most of the respondents who were WFH experienced some degree of stress (83.2%), with 15.1% reporting that WFH caused them significant stress.

### 3.1. Exploratory Factor Analysis (EFA)

The Kaiser–Meyer–Olkin measure of sample adequacy for BRS-M was 0.766, which was higher than the suggested value of 0.6 and indicated mediocrity [48]. Bartlett’s test of sphericity was significant (χ^2^_(15)_ = 334.3, *p* < 0.050) and specified that a relationship existed between at least some of the items, and the data were suitable for factor analysis. All initial communalities were above the threshold, with all loading factors above 0.5.

Two factors were extracted based on parallel analysis [42]. The first factor that was related to the three negatively worded items labelled as “Succumbing” (item 2, item 4, and item 6) explained 51.88% of the variance, and the second factor related to the three positively worded items labelled as “Resilience” (item 1, item 3, and item 5) explained 19.43 % of the variance (Table 1). The total variance of these two factors was 71.31%, which was higher than 50% [49]. The BRS-M exhibited good internal consistency, with Cronbach’s alpha coefficients for the subscales being 0.911 and 0.878, respectively.

### 3.2. Concurrent (Criterion) Validity of BRS-M

The results showed the concurrent validity of BRS-M, which was supported by significantly moderate negative correlations with all domains in the Malay Copenhagen Burnout Inventory (CBI-M) and Malay Depression, Anxiety, and Stress Scale (M-DASS-21), as shown in Table 2.

### 3.3. Confirmatory Factor Analysis (CFA)

Based on the results using Pratt’s importance measure matrix program, the following results of CFA were obtained. All six items demonstrated an outer loading greater than the 0.5 threshold. The subscales “Resilience” for positively phrased items and “Succumbing” for negatively phrased items had average variance extracted (AVE) values of 0.48 and 0.70, respectively, and all outer loadings of all items for two components were greater than 0.5.

The composite reliability (CR) for subscales “Resilience” and “Succumbing” were 0.73 and 0.87, respectively, which were both over 0.7 for this study. The findings thus demonstrated the existence of convergent validity and construct reliability for this study’s constructs. The HTMT method (Hetrotrait-Monotrait ratio of criteria) showed the presence of discriminant validity between the two subscales, which was 0.69 below the threshold of 0.85. Cross-loadings were also tested, and the results revealed that there were no cross-loaded items between the two components. CFA successfully replicated the two-factor structure from EFA, with sufficient internal consistency reliability (Cronbach’s α = 0.806 and McDonald’s omega, ω = 0.812) and a value of standardized root mean square residual (SRMR) of 0.031 (≤0.08 threshold), which is considered a good fit [50].

**Figure 1:**
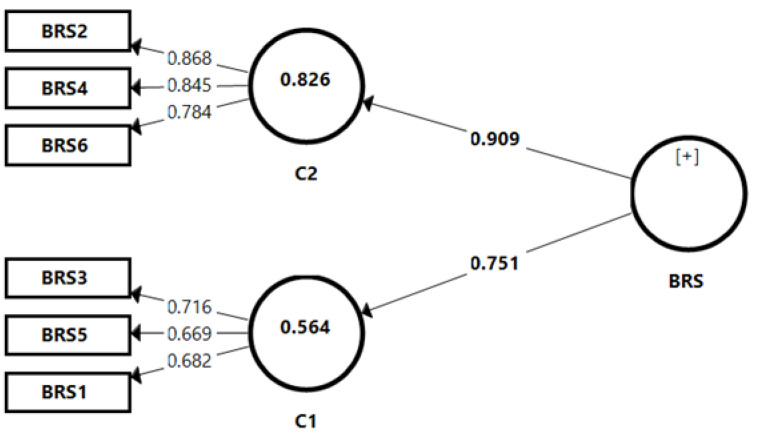
Output of Pratt’s Importance Measure Matrix Model.

The results of CFA using PLS-SEM for the convergent validity of two subscales are presented in Table 3. The Brief Resilience Scale Malay version is valid and reliable, as shown in the Figure 1.

### 3.4. Resilience Level among Non-Academic Staff

Overall, 70% of the non-academic staff had a normal resilience level based on the BRS-M (score of 3–4.3), and 22.5% of them were noted to have a low resilience level, with a BRS-M score of less than 3, while 7.4% were found to have a higher resilience level (score > 4.3).

### 3.5. Univariable and Multivariable Analysis on Factors Associated with BRS-M

The subscale “Succumbing” was found to be associated with marital status (*p* = 0.023), and “Resilience” had significant association with household income with *p* = 0.029. Both marital status (*p* = 0.010) and household income (*p* = 0.023) demonstrated significant association with BRS-M total score (Table 4).

Table 5 shows that the findings demonstrated the utility of the predictive model as significant, *F*(8,289) = 19.11, *R*^2^ = 0.346 (adjusted 0.328), *p* < 0.001. All of the predictors account for 32.8% of the shared variation and overlap between the variables in the model. The results showed that low-income group demonstrated a 0.299% lower resilience score (B = −0.299, 95% CI [−0.529, −0.070], t = 2.604, *p* = 0.011) as compared to higher income group.

## 4. Discussion

The present study aimed to translate and validate the Brief Resilience Scale (BRS) to the Malay language and is the first such study in Malaysia. The Malay version of the Brief Resilience Scale (BRS-M) was evaluated among non-academic staff working in a public university in Malaysia during the COVID-19 pandemic. A total of 98.3% of the respondents were working from home, and of those, 83.2% perceived working from home remotely as stressful. Hence, a valid and reliable instrument that measures resilience following a perceived stressful event among these workers is important, especially when they return to work.

Resilience has been highlighted as an essential element to overcome adversities, and therefore, it is considered important to validate the scale that has been widely used, namely the Brief Resilience Scale. Based on the exploratory factor analysis (EFA) and confirmatory factor analysis (CFA) studies, the six-item Malay version of the Brief Resilience Scale (BRS-M) yielded adequate properties for construct validity, concurrent and discriminant validity, and reliability. The dimensionality for BRS-M was carried out on a separate group of respondents to ensure “the measurement of items, their factors, and function are the same across two independent samples” [51].

The original BRS developed by Smith et. al exhibited a unidimensional construct based on EFA, but the underlying latent constructs were not verified with CFA [32]. The two-factor model has been discussed in the literature using CFA to verify the dimensionality of BRS supporting the two latent factors. BRS-M exhibited a two-factor model in which the first factor was labelled as “Resilience” and composed of positively worded items 1, 3, and 5. The second factor corresponded to “Succumbing”, with negatively worded items 2, 4, and 6. The two-factor model demonstrated by the BRS Malay version is similar to the analyses carried out in the Greek [52], Chinese [23], Polish [53], Spanish [24], and Romanian [54] versions. A consistently good fit was provided by the two-factor model and was considered more reliable than the unidimensional construct, as shown in this study. This outcome is explained by the wording effect, which led to item reversing and forced the items to evenly separate into positively worded and negatively worded items [52].

The results also demonstrated sufficient internal consistency reliability based on the Cronbach’s α and McDonald’s ω values. McDonald’s omega value has been reported to represent the strength of association between items and constructs for better estimates of reliability of the instrument as compared to Cronbach’s α [55]. BRS-M also demonstrated a good model fit by evidence of the SRMR value of less than 0.08. The AVE of the construct “Resilience” was less than 0.5 as compared to “Succumbing”, but according to Fornel and Larcker, the AVE of 0.4 is acceptable if the composite reliability is higher than 0.6, and the convergent validity of the construct is considered adequate [56]. The composite reliability (CR) for both factors were 0.872 and 0.731, respectively. CR is reported to be a preferred alternative to Cronbach’s alpha as a measure of reliability in a reflective model using the SEM-PLS approach [57].

BRS-M was analysed further based on the sociodemographic factors of the participants. The majority of the participants were married and belonged to the middle- and low-income groups. Marital status and household income were significantly associated with resilience, with low household income being a predictor of lower resilience. It is commonly seen among the administrative staff in the Malaysian public sector that they are represented by Malay women from middle- and low-income groups. This outcome demands an in-depth look at the well-being of employees who are already vulnerable to work-related stress and the demands by the organization for them to “bounce back” after returning to work.

Several limitations of this study were identified. First, this study targeted a population that was already affected by the COVID-19 pandemic and hence already in a state of significant vulnerability compounded by the fact that they were expected to maintain their productivity before pandemic by the respective organizations. The majority of the respondents were in the middle- to low-income group, women, married, and Malays. Second, the study was conducted in a single institution, which limits the generalization to other settings and cultural backgrounds. Third, the mode of data administration was online rather than face-to-face, with a lack of participation from the executive level of the non-academic staff, which may provide different results. Fourth, the timing of the assessment during lockdown may affect the reliability of the findings since the participants’ condition was unstable over time. Fifth, the sample consisted of 149 participants equally for EFA and CFA, and some studies have proposed a minimum of 300 to generate a stable factor loading and for the results to be generalizable [58,59].

## 5. Conclusions

The BRS-M exhibited a favourable reliability and construct validity and replicated a two-factor structure, and low resilience level demonstrated a significant association with the domains of burnout and psychological distress. The BRS-M can be applied in administrative populations to measure the effectiveness of interventions that promote adaptation, resilience, and recovery from stressful life events.

## Figures and Tables

**Table 1:** Factor analysis of Malay version of the Brief Resilience Scale (BRS-M).

Items	Factors	
	1 Succumbing	2 Resilience
Item 2 I have a hard time making it through stressful events. *Saya mempunyai kesukaran untuk mengharungi peristiwa yang menekankan.*	0.954	
Item 4 It is hard for me to snap back when something bad happens. *Sukar untuk saya kembali kepada keadaan sediakala apabila sesuatu yang buruk berlaku.*	0.842	
Item 6 I tend to take a long time to get over setbacks in my life. *Saya cenderung untuk mengambil masa yang lama untuk mengatasi halangan-halangan di dalam hidup saya.*	0.795	
Item 1 I tend to bounce back quickly after hard times. *Saya cenderung untuk bangkit kembali dengan kadar segera setelah melalui sesuatu tempoh yang sukar.*		0.975
Item 3 It does not take me long to recover from a stressful event. *Ianya tidak memerlukan saya masa yang lama untuk pulih daripada peristiwa yang menekankan.*		0.525
Item 5 I usually come through difficult times with little trouble. *Saya hampir tiada masalah apabila menghadapi situasi yang sukar.*		0.622
Eigenvalues	3.113	1.166
% of Variance	51.88	19.43
Cronbach’s α	0.911	0.878

**Table 2:** Pearson correlations between the BRS-M with CBI-M and M-DASS-21.

Scales		BRS-M
CBI-M	Personal	−0.449 **
	Work-related	−0.462 **
	Client-related	−0.373 **
M-DASS 21	Depression	−0.530 **
	Anxiety	−0.416 **
	Stress	−0.492 **

** *p* < 0.001; BRS-M, Malay Version of Brief Resilience Scale; CBI-M, Malay version of Copenhagen Burnout Inventory; M-DASS-21, Malay version of Depression, Anxiety, and Stress Scale-21.

**Table 3:** Cronbach α, average variance extracted (AVE) values, and composite reliability (CR) among the two constructs.

Constructs	Item	Outer Loading	Cronbach’s Alpha	Composite Reliability	Average Variance Extracted
Resilience C1	BRS3 BRS5 BRS1	0.716 0.669 0.682	0.448	0.731	0.475
Succumbing C2	BRS2 BRS4 BRS6	0.868 0.845 0.784	0.778	0.872	0.694

**Table 4:** Univariable analysis between BRS-M subfactor scores and BRS-M total score with sociodemographic factors (n = 298).

Variables	N (%)	Succumbing		Resilience		BRS-M Total Score	
		Mean (SD)	F/t Statistics (*p*-Value)	Mean (SD)	F/t Statistics (*p*-Value)	Mean (SD)	F/t Statistics (*p*-Value)
Age group							
18–35 36–45 46–60	110 (36.9) 123 (41.3) 65 (21.8)	3.25 (0.89) 3.45 (0.85) 3.37 (0.77)	F = 1.644 (0.195)	3.39 (0.70) 3.43 (0.67) 3.57 (0.65)	F = 1.500 (0.225)	3.32 (0.70) 3.44 (0.67) 3.47 (0.51)	F = 1.481 (0.230)
Gender							
Male Female	44 (14.8) 254 (85.2)	3.28 (0.86) 3.37 (0.85)	t = −0.658 (0.511)	3.48 (0.63) 3.44 (0.69)	t = 0.323 (0.747)	3.38 (0.63) 3.41 (0.65)	t = −0.261 (0.794)
Race							
Malay Chinese/Indian/ Others	268 (89.9) 30 (10.1)	3.36 (0.85) 3.36 (0.88)	t = 0.016 (0.987)	3.45 (0.66) 3.42 (0.81)	t = 0.233 (0.816)	3.41 (0.64) 3.39 (0.71)	t = 0.133 (0.895)
Marital status							
Single Married	68 (22.8) 230 (77.2)	3.15 (0.87) 3.42 (0.84)	t = −2.294 (0.023) *	3.30 (0.80) 3.49 (0.63)	t = −1.843 (0.068)	3.22 (0.74) 3.46 (0.61)	t = −2.604 (0.010) *
Education level							
Secondary school Diploma Degree	78 (26.2) 97 (32.6) 123 (41.3)	3.24 (0.85) 3.47 (0.85) 3.35 (0.85)	F = 1.699 (0.185)	3.50 (0.59) 3.36 (0.63) 3.49 (0.76)	F = 1.212 (0.299)	3.37 (0.56) 3.42 (0.63) 3.42 (0.72)	F = 0.207 (0.813)
Household income ^#^							
B40 M40 T20	131 (44.0) 140 (47.0) 27 (9.1)	3.27 (0.80) 3.39 (0.89) 3.63 (0.80)	F = 2.142 (0.119)	3.35 (0.67) 3.49 (0.66) 3.70 (0.75)	F = 3.588 (0.029) *	3.31 (0.59) 3.44 (0.67) 3.67 (0.73)	F = 3.801 (0.023) *
Working from home (WFH)							
No Yes	5 (1.7) 293 (98.3)	3.40 (0.89) 3.36 (0.85)	t = 0.112 (0.911)	2.87 (1.04) 3.46 (0.67)	t = −1.946 (0.053)	3.13 (0.94) 3.41 (0.64)	t = −0.940 (0.348)

SD, standard deviation; F, one-way ANOVA test; t, independent *t*-test; *, ≤ 0.05; ^#^, T20 (above MYR 9619 or top 20%), M40 (MYR 4360–MYR 9619 or middle 40%), and B40 (less than MYR 4360 or below 40%).

**Table 5:** Multivariable analysis on factors associated with BRS-M total score (n = 298).

Factors	Crude B (95% CI)	*p*-Value	Adjusted B (95% CI)	*p*-Value
Marital status				
Single	−0.231 (−0.406, −0.056)	0.010 *	−0.095 (−0.250, 0.059)	0.227
Married				
Household income				
B40	−0.355 (−0.623, −0.088)	0.009 *	−0.299(−0.529, −0.070)	0.011 *
M40	−0.228 (−0.494,0.038)	0.092	−0.179 (−0.402, 0.044)	0.115
T20				
Personal burnout	−0.016 (−0.020, −0.012)	<0.001 *	−0.002 (−0.008, 0.003)	0.405
Work-related burnout	−0.017 (−0.021, −0.013)	<0.001 *	−0.004 (−0.010,0.002)	0.214
Client-related burnout	−0.016 (−0.020, −0.011)	<0.001 *	−0.004 (−0.009, 0.001)	0.105
Depression	−0.100 (−0.118, −0.082)	<0.001 *	−0.047 (−0.077, −0.016)	0.003 *
Anxiety	−0.089 (−0.111, −0.067)	<0.001 *	0.005 (−0.027, 0.037)	0.758
Stress	−0.092 (−0.110, −0.073)	<0.001 *	−0.029 (−0.062, 0.004)	0.082

*, ≤ 0.05.

## Data Availability

The data presented in this study are available on request from the corresponding author. The data are not publicly available due to confidentiality.

## References

[1] Xiong J., Lipsitz O., Nasri F., Lui L.M.W., Gill H., Phan L., Chen-Li D., Iacobucci M., Ho R., Majeed A. (2020). Impact of COVID-19 pandemic on mental health in the general population: A systematic review. J. Affect. Disord..

[2] Clemente-Suárez V.J., Martínez-González M.B., Benitez-Agudelo J.C., Navarro-Jiménez E., Beltran-Velasco A.I., Ruisoto P., Diaz Arroyo E., Laborde-Cárdenas C.C., Tornero-Aguilera J.F. (2021). The Impact of the COVID-19 Pandemic on Mental Disorders. A Critical Review. Int. J. Environ. Res. Public Health.

[3] Wang C., Pan R., Wan X., Tan Y., Xu L., Ho C.S., Ho R.C. (2020). Immediate psychological responses and associated factors during the initial stage of the 2019 coronavirus disease (COVID-19) epidemic among the general population in China. Int. J. Environ. Res. Public Health.

[4] Tang K.H.D. (2022). Movement control as an effective measure against Covid-19 spread in Malaysia: An overview. J. Public Health.

[5] Bick A., Blandin A., Mertens K. (2020). Work from Home after the COVID-19 Outbreak.

[6] Lucey C.R., Johnston S.C. (2020). The transformational effects of COVID-19 on medical education. JAMA.

[7] Alwi N.H., Osman Z., Ismail Z., Khan B.N.A. (2022). Higher Educations Employees Work From Home Productivity during The COVID-19 Outbreak: The Role Of Motivation As Mediator. Asian J. Manag. Entrep. Soc. Sci..

[8] Shockley K.M., Allen T.D. (2012). Motives for flexible work arrangement use. Community Work. Fam..

[9] Salim N., Chan W.H., Mansor S., Nazira Bazin N.E., Amaran S., Mohd Faudzi A.A., Zainal A., Huspi S.H., Jiun Hooi E.K., Shithil S.M. (2020). COVID-19 epidemic in Malaysia: Impact of lockdown on infection dynamics. medrxiv.

[10] Tull M.T., Edmonds K.A., Scamaldo K.M., Richmond J.R., Rose J.P., Gratz K.L. (2020). Psychological outcomes associated with stay-at-home orders and the perceived impact of COVID-19 on daily life. Psychiatry Res..

[11] Chen Z. (2021). Influence of Working From Home During the COVID-19 Crisis and HR Practitioner Response. Front. Psychol..

[12] Dockery M., Bawa S. (2020). Working from Home in the COVID-19 Lockdown.

[13] Windle G., Bennett K.M., Noyes J. (2011). A methodological review of resilience measurement scales. Health Qual. Life Outcomes.

[14] Zabaniotou A. (2020). A systemic approach to resilience and ecological sustainability during the COVID-19 pandemic: Human, societal, and ecological health as a system-wide emergent property in the Anthropocene. Glob. Transit..

[15] Manning L., Ferris M., Rosario C.N., Prues M., Bouchard L. (2019). Spiritual resilience: Understanding the protection and promotion of well-being in the later life. J. Relig. Spirit. Aging.

[16] Pietrzak R.H., Southwick S.M. (2011). Psychological resilience in OEF–OIF Veterans: Application of a novel classification approach and examination of demographic and psychosocial correlates. J. Affect. Disord..

[17] Salata K.-D., Yiannakou A. (2020). The Quest for Adaptation through Spatial Planning and Ecosystem-Based Tools in Resilience Strategies. Sustainability.

[18] Friedli L., WHO Regional Office for Europe (2009). Mental Health, Resilience and Inequalities.

[19] Holling C.S. (1973). Resilience and Stability of Ecological Systems. Annu. Rev. Ecol. Syst..

[20] Wake L. (2008). Neurolinguistic Psychotherapy: A Postmodern Perspective.

[21] Werner E.E., Smith R.S. (1992). Overcoming the Odds: High Risk Children from Birth to Adulthood.

[22] Namok C., Stephen M.L., Hart J.M., Hongryun W. (2019). Further Validation of the Brief Resilience Scale from a Korean College Sample. J. Asia Pac. Couns..

[23] Fung S.-f. (2020). Validity of the Brief Resilience Scale and Brief Resilient Coping Scale in a Chinese Sample. Int. J. Environ. Res. Public Health.

[24] Rodríguez-Rey R., Alonso-Tapia J., Hernansaiz-Garrido H. (2016). Reliability and validity of the Brief Resilience Scale (BRS) Spanish Version. Psychol. Assess..

[25] Haktanir A., Lenz A., Can N., Watson J. (2016). Development and Evaluation of Turkish Language Versions of three Positive Psychology Assessments. Int. J. Adv. Couns..

[26] Amat S., Subhan M., Jaafar W.M.W., Mahmud Z., Johari K.S.K. (2014). Evaluation and Psychometric Status of the Brief Resilience Scale in a Sample of Malaysian International Students. Asian Soc. Sci..

[27] Boateng G.O., Neilands T.B., Frongillo E.A., Melgar-Quiñonez H.R., Young S.L. (2018). Best practices for developing and validating scales for health, social, and behavioral research: A primer. Front. Public Health.

[28] Zou G., Shen X., Tian X., Liu C., Li G., Kong L., Li P. (2016). Correlates of psychological distress, burnout, and resilience among Chinese female nurses. Ind. Health.

[29] Campbell-Sills L., Forde D.R., Stein M.B. (2009). Demographic and childhood environmental predictors of resilience in a community sample. J. Psychiatr. Res..

[30] Beutel M.E., Glaesmer H., Decker O., Fischbeck S., Brähler E. (2009). Life satisfaction, distress, and resiliency across the life span of women. Menopause.

[31] Tsang S., Royse C.F., Terkawi A.S. (2017). Guidelines for developing, translating, and validating a questionnaire in perioperative and pain medicine. Saudi J. Anaesth..

[32] Smith B.W., Dalen J., Wiggins K., Tooley E., Christopher P., Bernard J. (2008). The brief resilience scale: Assessing the ability to bounce back. Int. J. Behav. Med..

[33] Nunnally J., Bernstein I. (1994). Psychometric Theory.

[34] DOSM (2020). Pendapatan Dan Perbelanjaan Isi Rumah M40 Dan B40 Mengikut NegerI.

[35] Smith B.W., Epstein E.M., Ortiz J.A., Christopher P.J., Tooley E.M. (2013). The foundations of resilience: What are the critical resources for bouncing back from stress?. Resil. Child. Adolesc. Adults Transl. Res. Pract..

[36] Kristensen T., Borritz M., Villadsen E., Christensen K. (2005). The Copenhagen Burnout Inventory: A new tool for the assessment of burnout. Work. Stress.

[37] Andrew Chin R.W., Chua Y.Y., Chu M.N., Mahadi N.F., Wong M.S., Yusoff M.S.B., Lee Y.Y. (2017). Investigating validity evidence of the Malay translation of the Copenhagen Burnout Inventory. J. Taibah Univ. Med. Sci..

[38] Antony M.M., Bieling P.J., Cox B.J., Enns M.W., Swinson R.P. (1998). Psychometric properties of the 42-item and 21-item versions of the Depression Anxiety Stress Scales in clinical groups and a community sample. Psychol. Assess..

[39] Musa R., Fadzil M.A., Zain Z. (2007). Translation, validation and psychometric properties of Bahasa Malaysia version of the Depression, Anxiety and Stress Scale (DASS). ASEAN J. Psychiatry.

[40] Kim H.Y. (2013). Statistical notes for clinical researchers: Assessing normal distribution (2) using skewness and kurtosis. Restor. Dent. Endod..

[41] Lorenzo-Seva U., Ferrando P.J. (2006). FACTOR: A computer program to fit the exploratory factor analysis model. Behav. Res. Methods.

[42] Horn J.L. (1965). A Rationale and Test for the Number of Factors in Factor Analysis. Psychometrika.

[43] Ringle C.M. (2005). SmartPLS 2.0 (M3). http://www.smartpls.de.

[44] Cronbach L.J. (1951). Coefficient alpha and the internal structure of tests. Psychometrika.

[45] Werts C.E., Linn R.L., Jöreskog K.G. (1974). Intraclass Reliability Estimates: Testing Structural Assumptions. Educ. Psychol. Meas..

[46] Hair J.F., Hult G.T.M., Ringle C.M., Sarstedt M. (2021). A Primer on Partial Least Squares Structural Equation Modeling (PLS-SEM).

[47] Bentler P.M. (1990). Comparative fit indexes in structural models. Psychol. Bull..

[48] Kaiser H.F. (1974). An index of factorial simplicity. Psychometrika.

[49] Streiner D.L. (1994). Figuring out factors: The use and misuse of factor analysis. Can. J. Psychiatry.

[50] Hu L.-t., Bentler P.M. (1998). Fit indices in covariance structure modeling: Sensitivity to underparameterized model misspecification. Psychol. Methods.

[51] Brown T.A. (2015). Confirmatory Factor Analysis for Applied Research.

[52] Kyriazos T., Stalikas A., Prassa K., Galanakis M., Yotsidi V., Lakioti A. (2018). Psychometric Evidence of the Brief Resilience Scale (BRS) and Modeling Distinctiveness of Resilience from Depression and Stress. Psychology.

[53] Konaszewski K., Niesiobędzka M., Surzykiewicz J. (2020). Validation of the Polish version of the Brief Resilience Scale (BRS). PLoS ONE.

[54] Macovei C.M. (2015). The Brief Resilience Scale—A Romanian-Language Adaptation.

[55] Dunn T.J., Baguley T., Brunsden V. (2014). From alpha to omega: A practical solution to the pervasive problem of internal consistency estimation. Br. J. Psychol..

[56] Fornell C., Larcker D.F. (1981). Evaluating structural equation models with unobservable variables and measurement error. J. Mark. Res..

[57] Brunner M., Süβ H.-M. (2005). Analyzing the reliability of multidimensional measures: An example from intelligence research. Educ. Psychol. Meas..

[58] Clark L.A., Watson D., Kazdin A.E. Constructing Validity: Basic Issues in Objective Scale Development. Methodological Issues and Strategies in Clinical Research.

[59] MacCallum R., Browne M., Sugawara H., Modeling P. (1996). Power Analysis and Determination of Sample Size for Covariance Structure Modeling. Psychol. Methods.

